# Impact of the COVID-19 Pandemic on Antibiotic Prescriptions at the University Clinical Dentistry Center of Kosovo

**DOI:** 10.3390/antibiotics14040405

**Published:** 2025-04-15

**Authors:** Agon Hoti, Ivana Sutej, Arianit Jakupi

**Affiliations:** 1 Department of Pharmacy, University of Business and Technology, 10000 Pristina, Kosovo; 2 Department of Pharmacology, School of Dental Medicine, University of Zagreb, 10000 Zagreb, Croatia; 3 MediTech Shpk, 10000 Pristina, Kosovo; arianiti@gmail.com

**Keywords:** COVID-19, antibiotics, dental care, antimicrobial resistance, University Dental Clinical Center of Kosovo, prescription trends, antibiotic stewardship

## Abstract

**Background:** The COVID-19 pandemic led to significant disruptions in healthcare services worldwide, including dental care, resulting in increased reliance on antibiotics as a substitute for in-person dental interventions. **Aim:** This study aimed to examine the prescription of different classes of antibiotics at the University Dental Clinical Center of Kosovo during the COVID-19 pandemic and compare it with prescription trends from the pre-pandemic period. **Methodology:** This study analyzed antibiotic prescription patterns at the University Dental Clinical Center of Kosovo (UCDCK) from 2019 to 2022, focusing on dental patients. Data were collected using a standardized form and a review of electronic health records. Descriptive statistics were used to examine trends, which were compared to data from other countries facing similar healthcare disruptions. The data is organized into quartiles, allowing for a comprehensive understanding of the distribution and trends in antibiotic usage over the four-year period. **Results:** The study revealed a significant increase in the prescription of broad-spectrum antibiotics, including amoxicillin, clavulanic acid, clindamycin, and metronidazole, during the pandemic period. Data collected from the university dentistry center showed that the highest frequency of antibiotic prescriptions occurred in 2021 (27.6%), while the lowest was in 2022 (22.8%). Metronidazole (39.4%) and amoxicillin with clavulanic acid (38.5%) were the most frequently prescribed antibiotics, together accounting for the majority of prescriptions. Amoxicillin alone represented 18.2%, with other antibiotics prescribed at significantly lower rates. These findings highlight the reliance on these antibiotics for dental treatments and underscore the importance of monitoring prescription trends to optimize usage and minimize resistance risks. **Conclusions:** The study highlights the impact of the COVID-19 pandemic on dental antibiotic prescription practices in Kosovo, revealing a concerning increase in broad-spectrum antibiotic use. This underscores the need for improved antibiotic stewardship in dental settings, particularly during public health crises, to prevent the exacerbation of antimicrobial resistance. Ensuring continued access to routine dental care and developing robust protocols for antibiotic prescription during emergencies are essential to mitigate the long-term public health impacts of increased antibiotic use.

## 1. Introduction

In a pre-2020 world, health care providers would diligently monitor the prescription and consumption of antibiotics due to the concerning trend of antimicrobial resistance. The COVID-19 pandemic brought unprecedented challenges to healthcare systems across the globe, revealing vulnerabilities in many sectors, including dentistry. This disease has been declared a state of emergency in public. These vulnerabilities were echoed in global reports that assessed the pandemic’s effect on various health professions [1]. Worldwide, over 100 million people were infected on 28 January 2020, and more than 2.15 million people have died since then [2]. 

The pandemic also led to social, environmental, and behavioral changes that indirectly affected healthcare demand [3]. However, the COVID-19 pandemic upended this restive status quo in two principal ways [4]. First, the pandemic transformed dentistry into an emergency-only specialty that resulted in patients, even those requiring non-urgent treatments, not receiving adequate care and clinicians being pigeon-holed into relying on antibiotics alone. This imbalance puts a phenomenal strain on dental practices and the healthcare system as a whole. The other stark change was that during the pandemic (and lockdowns that accompanied it), people were forced to self-quarantine, and that pushed people to self-medicate with broad-spectrum antibiotics to sidestep the cumbersome process of booking an appointment with a practitioner. This culminated in a spike in misuse of antibiotics and inappropriate prescribing to a point where most practitioners had to be wary of the shambles the healthcare system had transformed into.

In this new chaotic world, emerging countries, especially Kosovo, were not spared. Kosovo, like every other country, is affected by global issues such as the pandemic. Regular dental services were impacted heavily [5]. That said, this study aims to analyze how dental care and other services galloped forward post-pandemic and how that affected the prescription trends of antibiotics in Kosovo.

Similar limitations in the effectiveness of alternative treatments, like CPAP for respiratory conditions, have also been observed during COVID-19 [6].

It is important to analyze these trends to plan future antibiotic stewardship programs effectively, thereby improving the management of antibiotics and their use in dentistry.

## 2. Results

The analysis reveals significant fluctuations in the overall use of antibiotics and the specific classes administered during the study period. Data collected from the university dental center were analyzed comprehensively, and the findings are detailed below to highlight trends, variations, and prescribing patterns.

According to the research, patient visits fluctuated from 2019 to 2022. The volume of patients was highest in 2019 and 2021, whereas a drop was seen in 2020 when the COVID-19 restrictions were the hardest. Furthermore, there was a drop in 2022, which can be attributed to the increase in telehealth and the normalization of healthcare demands, as Table 1 shows.

In 2021, the number of patients recovered as the healthcare system adjusted to pandemic conditions with new protocols and worked on the backlog of needed treatments from the preceding year. In contrast, 2022 showed a drastic decrease in the number of patients, which might be linked to the reduction of healthcare needs in the aftermath of the pandemic and greater use of remote healthcare technologies, which would decrease the necessity for physical consultations.

The highest prescription frequency is observed in 2021, with a maximum percentage of 27.6%, while the lowest frequency is recorded in 2022, at 22.8%. These fluctuations reflect the broader impacts of the COVID-19 pandemic and other healthcare dynamics, highlighting changing patterns in antibiotic use.

### Detailed Antibiotic Prescribing Trends

The frequency of antibiotic cases recorded over four years reveals several patterns, as presented in Table 1. The increase in cases observed in 2021, 482 cases accounting for 27.6%, was the highest frequency recorded over the years. The increase from the previous year was significant, and the lowest frequency was recorded in 2022, with 399 cases, equivalent to 22.8%. The frequency recorded reflects a considerable decrease (χ^2^ = 23.56, *p* < 0.001) as the dental healthcare system started functioning normally.

In 2021, patient numbers rebounded as the healthcare system adapted to pandemic conditions, implemented new protocols, and addressed the residual load of deferred treatments from the previous year. However, 2022 experienced a significant decline in patient numbers, which could be associated with the normalization of healthcare demand post-pandemic and increased adoption of telehealth services, reducing the need for in-person visits.

**Figure 1:**
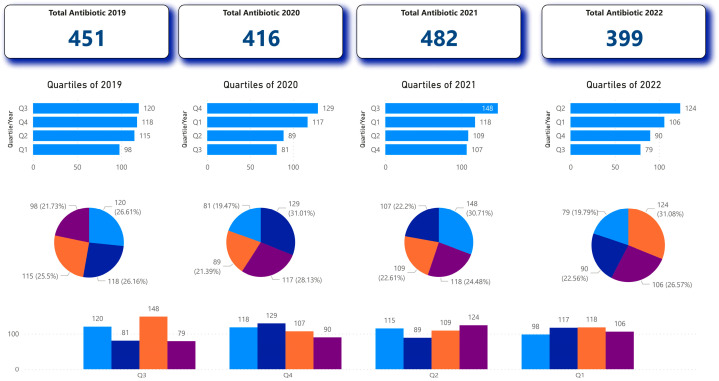
Quartile analysis of antibiotic prescription in the years 2019–2022.

As presented in Figure 1, a detailed analysis of antibiotic prescription patterns at the University Dental Clinical Center of Kosovo (UCDCK) from 2019 to 2022. The data is organized into quartiles, allowing for a comprehensive understanding of the distribution and trends in antibiotic usage over the four-year period.

In 2019, the total number of antibiotics prescribed was 451, with the quartile values showing a relatively even distribution across the different levels. The following year, 2020, saw a slight decrease in the total number of antibiotics to 416, with the lower quartiles (Q1 and Q2) showing a reduction in prescription volumes compared to the previous year.

The year 2021 marked a significant shift, with the total number of antibiotics prescribed increasing to 482, the highest during the study period. This is reflected in the quartile data, where the upper quartile (Q3) reached 148, indicating a substantial rise in the frequency of higher-volume antibiotic prescriptions.

In 2022, the total number of antibiotics prescribed decreased to 399, the lowest among the four years analyzed. The quartile values also show a more even distribution, with the upper quartile (Q3) at 124 and the lower quartiles (Q1 and Q2) at 106 and 90, respectively.

As we can see from Table 2, regarding antibiotic prescriptions, the study’s results suggest that the most prescribed antibiotics were metronidazole and amoxicillin with clavulanic acid, which covered 77.9% of all prescriptions. Metronidazole was used far more frequently than other antibiotics, which is statistically significant (χ^2^ = 19.82, *p* < 0.001), emphasizing its vital place in managing dental infections.

During the more severe phases of the pandemic in 2020 and 2021, we saw an increase in the use of metronidazole (39.4%). This highlights its use as a first line of defense when in-person treatment was too tricky.

The usage of amoxicillin and clavulanic acid remained consistent. Prescriptions slightly increased in 2021 (38.5%), only to drop again in 2022. In comparison, amoxicillin accounts for 18.2% of prescriptions separately, demonstrating its importance in hospital treatment settings.

According to the the data presented in Table 2, metronidazole was prescribed in 688 cases (39.4% of total prescriptions), making it the most-used antibiotic. Amoxicillin with clavulanic acid was followed closely by 673 prescriptions (38.5%).

When amoxicillin is considered, the total prescriptions for these antibiotics rise to 992, which is over 75% of the total cases.

## 3. Discussion

The usage of antibiotics during the COVID-19 pandemic at the University Clinical Dentistry Center of Kosovo reveals that dental practitioners had little choice other than to follow the global trend. The data suggests that there was an increased use of antibiotics because of restricted patient attendance along with the in-pandemic antibiotic trends. Metronidazole and amoxicillin with clavulanic acid were the most used medicines in the study, which agrees with global studies conducted during the pandemic.

The disproportionate amount of metronidazole and amoxicillin is most likely due to the common nature of dental infections, which require a quick, effective solution, and these two fit the bill perfectly. More importantly, this data emphasizes the need for continuous promotion and education on responsible antibiotic use to combat prescription overreach and stave off growing resistance.

The rise in antibiotic prescriptions in 2021 was driven by postponed dental treatments due to the pandemic, while the decline in 2022 marked a return to maintenance-based care. These shifts reflect broader healthcare trends and potential impacts on antimicrobial resistance.

Selective use of azithromycin, clindamycin, and erythromycin suggests targeted prescribing, with a notable but unproven increase in azithromycin for COVID-19 superinfections. The high use of metronidazole and amoxicillin with clavulanic acid highlights infection control needs but also raises concerns about resistance, emphasizing the need for careful prescribing.

Additionally, local antibiotic applications, such as clindamycin used to prevent dry socket after molar extractions, have been explored in Kosovo dental clinics [7].

Multiple international studies highlight a noteworthy rise in antibiotic utilization in many countries throughout the pandemic. In England, Shah et al. [8] and in Scotland, Duncan [9] reported a 25% and 49% increase in the use of antibiotics, respectively, after partitioning dental care services. These findings concur with other studies conducted in France (Bara [10]), Australia (Mian [11]), and Norway (Tousi [12]), where there was an increase in the use of the medicament.

Hungary also reported changes in dental antibiotic redemption trends during this period [13].

Similar increases were observed in Croatia’s emergency dental services [14].

Nonetheless, Kitano [15] in Canada and Immel [16] in Ontario noted a drop in antibiotic prescription rates (31.2%), which was correlated with the imposition of strict public health measures and improved management of dental services. This implies that local policies and the structure of health systems affected the degree of antibiotic use during the pandemic.

In Kosovo, studies conducted by Hoti,A [17] and Tolaj,I [18,19] noted sharp increases in the prescription of wide-spectrum antibiotics, including amoxicillin with clavulanic acid, clindamycin, and metronidazole. This increase emphasizes the struggles of the dental care system in controlling severe cases during the pandemic and the reliance on drug therapy because of the absence of in-person care.

Pre-pandemic studies had already highlighted widespread use of broad-spectrum antibiotics in Kosovo’s hospital settings, including among pediatric and adult patients [20].

The variance trends in antibiotic utilization across various countries are a function of multiple variables, such as healthcare accessibility, national guidelines on prescribing, and the level of COVID pandemic restrictions. While Kosovo experienced an increase in antibiotic use owing to insufficient dental services, Canada, on the other hand, registered a decrease, which was made possible through telehealth services and monitored prescription policies. These differences emphasize the need for systematic public health intervention to regulate prescription practices. The economic burden of COVID-19 on healthcare institutions further complicated effective dental care delivery [21].

Of particular concern is the increase in antibiotic consumption during the pandemic and, along with it, antimicrobial resistance (AMR). Krasniqi [22] and Mustafa,L [23]’s studies point out the overuse of sole antibiotics among hospitalized patients suffering from COVID-19 and the long-term effects of such prescription sobriety habits [1,24,25].

In the same way, Sulis [26] and Subramanya [27] have mentioned that the pandemic-related surge in antibiotic use worsened the AMR situation worldwide. Šutej [17] in Croatia claimed a 39.3% growth in azithromycin prescriptions, which stresses the worldwide phenomenon of excessive antibiotic use in the absence of active surgical management in dental practice.

Considering this, it is essential to implement antibiotic stewardship programs to combat the inappropriate use of drugs and manage antimicrobial resistance problems.

These topics were also addressed during recent international dental congresses in Zagreb, where the emphasis was placed on adapting protocols for antibiotic prescribing during pandemics [24,28].

### Limitations and Strengths of the Study

The study provides valuable insights into the fluctuations in antibiotic prescribing patterns at the University Clinical Dentistry Center of Kosovo during the COVID-19 pandemic, highlighting the impact of the pandemic on dental care and medication use. The detailed breakdown of the most commonly prescribed antibiotics, such as metronidazole and amoxicillin with clavulanic acid, offers essential information about the infection control needs in dentistry during the pandemic. Additionally, the study’s findings align with global trends observed in other countries, suggesting that the increased use of certain antibiotics was widespread during the COVID-19 pandemic, likely due to restricted patient attendance and the need for quick, practical solutions.

While this study provides valuable insights into antibiotic prescribing trends, several limitations must be acknowledged. The analysis relies on retrospective data, which may be subject to inconsistencies in record-keeping. The study does not assess patient adherence to prescribed antibiotic regimens, which could influence treatment outcomes. External factors, such as government policies and public health interventions, were not explicitly analyzed, yet they likely played a significant role in shaping prescribing behaviors.

This analysis of various studies worldwide focuses on the most important aspects of the patterns of prescribing antibiotics for use in dentistry during the pandemic (Table 3).

The results presented in Table 3 indicate that antibiotic prescribing patterns changed during the COVID-19 period. This demonstrates how healthcare services worldwide manage issues resulting from limited access to conventional routines for attending to dental hygienic procedures.

Most countries saw a marked rise in the prescribing of antibiotics after suspending elective dental procedures. For example, Shah et al. [8] in England and Duncan [9] in Scotland reported increases in antibiotic prescriptions of 25% and 49%, respectively. The increase in prescriptions was mainly due to the increased use of amoxicillin, clindamycin, and metronidazole. In the same way, Šutej [17] in Croatia documented a 39.3% increase in prescriptions for azithromycin, which indicates excessive dependency on antibiotics in the absence of necessary surgical interventions.

Although an increase in the use of antibiotics was noted in the more significant part of the world, some countries reported a decrease owing to more stringent measures of protection against the pandemic. According to Kitano [15], this was the case in Ontario, Canada, which saw a 31.2% decrease in prescriptions. This suggests dental care was managed remotely quite effectively. Similarly, Immel [16] and Rodríguez-Fernández [34] observed irregularities in prescribing, where some areas reduced antibiotic use at first, then increased it after the restoration of the dental practices.

The prescription of broad-spectrum antibiotics increased, especially amoxicillin with clavulanate, clindamycin, and metronidazole in Kosovo along with other nations, as reported by Hoti, A and Tolaj, I [17,18,19]. A spike in antibiotic use statistics by 30% was reported from 2020 to 2021. This increase highlights the dental healthcare sector’s struggles during the pandemic.

Furthermore, local studies revealed inconsistencies in selecting first-choice antibiotics for dental infections, emphasizing the need for standardized prescribing guidelines [51].

The outcomes support a global policy framework articulated by organizations like the World Health Organization (WHO) and the European Centre for Disease Prevention and Control (ECDC), emphasizing the importance of improved antibiotic stewardship in dentistry. In Kosovo, ICU patients hospitalized with COVID-19 were frequently administered antibiotics, as shown by Mustafa et al. [18]. Canada and Norway have successfully addressed the issue of antibiotic overuse by implementing real-time monitoring systems for dental antibiotic prescriptions. Similar strategies could be adopted in Kosovo to help reduce the reliance on broad-spectrum antibiotics. Additionally, national targets for antibiotic prescribing in dental treatment should be reinforced, and stricter regulations must be introduced to mitigate the risks of overprescribing.

A detrimental consequence of the abuse of antibiotics during the pandemic is the rapid development of antimicrobial resistance (AMR). As Krasniqi and Mustafa, L reported, spectrum antibiotic use during the hospitalization of COVID-19 patients was on the higher side [22,23]. This puts us at risk of developing long-term resistance.

In the same breath, but on a more alarming scale, Sulis (and Subramanya [26,27]) explain how the cessation of medical services and rampant usage of antibiotics have tremendously increased AMR. From there, it can be concluded that there is a need for much stricter antibiotic prescription rules.

These findings reiterate and strengthen the critical need to formulate broader policies on antibiotic prescribing in dentistry during a public health emergency.

Improving monitoring systems for antibiotic prescribing in real time in dental clinics.

Challenging the status quo of prescribing antibiotics and using enabling treatment methods.

Considering international best practices, stricter regulations on antibiotic prescriptions in Kosovo can potentially reduce the risks associated with antimicrobial resistance and enhance patient safety.

Other international research supports the finding that the COVID-19 pandemic affected dental antibiotic prescribing. In Croatia, longitudinal studies have noted some changes in the quality of prescriptions and some consumption over this period [38,42]. Similarly, Canadian public dental clinics observed changes in the volume of services and prescriptions [32]. Spain experienced a cycle of increased and decreased antibiotic prescribing, particularly among dentists in Galicia [34], and Italian hospitals observed an increase in resistance among Pseudomonas aeruginosa isolates [43]. In Norway, there were also some changes in antibiotic prescribing by dentists, which is now common in Europe [12].

Further international research has proven these observations, emphasizing changes related to the utilization of dental antibiotics, prescribing activities, and antimicrobial resistance during the COVID-19 pandemic [19,26,27,36,39,52,53,54].

## 4. Methodology

This study performed a retrospective quantitative analysis of antibiotic prescription patterns at the University Dental Clinical Center of Kosovo (UCDCK) from 2019 to 2022, focusing on dental patients who received antibiotics. The sample included records with complete prescription details, while incomplete records or those not linked to confirm dental infection diagnoses were excluded. Data were collected using a standardized patient form based on the Point Prevalence Study (PPS) framework, covering four time periods: pre-pandemic (January 2019–December 2019), pandemic onset (January 2020–December 2020), peak pandemic (January 2021–December 2021), and post-pandemic (January 2022–December 2022). A targeted search within UCDCK’s electronic health record system was conducted to extract relevant data, which was rigorously reviewed for accuracy and completeness. Descriptive statistical methods were then used to analyze trends in antibiotic usage, with findings compared to data from other countries facing similar healthcare disruptions, organized by geographic region, antibiotic types, and prescribing practices to provide a broader context for Kosovo’s trends.

## 5. Conclusions

The objective of this study was to determine the influence of the pandemic on the patterns of antibiotic prescriptions and how the changes in dental care services impacted the patterns in the use of various antibiotics. According to the results, there was a notable increase in the consumption of antibiotics during the COVID-19 period because of the dental care restriction in conjunction with the substitution of many dental procedures with medications. The data evidence indicates a disturbing increase in broad-spectrum antibiotics, namely metronidazole and amoxicillin with clavulanic acid, during the pandemic. The increase in the level of antibiotic prescriptions, reaching its peak in 2021, emphasizes the inability of the dental care system to keep severe cases in check and greater reliance on medication treatment due to restricted face-to-face services. The trend reflects the world scenario, where several countries recorded increased antibiotic use amid the pandemic due to the scarcity of regular dental facilities.

Overusing certain antibiotics is a cause of grave concern over the development of antimicrobial resistance. Hence, a pressing need is for increased antibiotic stewardship in dentistry, particularly during public health crises. Implementing effective antibiotic stewardship programs, improving surveillance systems, and promoting judicious prescribing are essential to tackle the challenges posed by the pandemic and prevent further propagation of antimicrobial resistance.

## Figures and Tables

**Table 1:** Number of antibiotic prescriptions in the years 2019–2022.

Year		Frequency	Percent	Cumulative Percent	Number of Patients over the Years
Valid	2019	451	25.8	25.8	400
	2020	416	23.8	49.6	370
	2021	482	27.6	77.2	400
	2022	399	22.8	100.0	300
	Total	1748	100.0		1470

**Table 2:** Frequency of antibiotics prescribed.

Name	Frequency	Percent
Amoxicillin and Clavulanic Acid	673	38.5
Amoxicillin	319	18.2
Ampicillin	1	0.1
Azithromycin	8	0.5
Cefalexin	6	0.3
Ciprofloxacin	3	0.2
Clindamycin	29	1.7
Erythromycin	20	1.1
Metronidazole	688	39.4

**Table 3:** Literature review findings of antibiotic prescriptions per patient.

Study Reference and Country	Key Findings	Prescription Trends/Antibiotics Used	Methodology
Shah et al. [8]	25% increase in antibiotic use due to limited dental access	Amoxicillin, Clindamycin	NHS Business Services Authority data analysis (2018–2020)
Duncan [9]	49% increase in prescriptions due to suspended routine dental care	Amoxicillin, Metronidazole	Public Health Scotland national prescribing data and online dentist survey
Bara [10]	Increased prescriptions among elderly patients and reduced pediatric use	Penicillin, Amoxicillin	French National Health Data System (2019–2020)
Mian [11]	Rise in prescriptions of amoxicillin with clavulanic acid	Amoxicillin + Clavulanic Acid	PBS data from January-June 2019 and 2020
Tousi [12]	Reversed downward trend in antibiotic use during COVID-19	Penicillin, Amoxicillin	Norwegian Prescription Register (2016–2021)
Šutej [17]	39.3% increase in azithromycin prescriptions	Azithromycin, Amoxicillin	Croatian Health Insurance Fund (2015–2020)
Krasniqi [22]	High dependency on pharmacological treatment with analgesics such as ibuprofen	Amoxicillin, Ibuprofen	Cross-sectional study on 55 severe COVID-19 patients
Hoti, A. [17]	Overuse of broad-spectrum antibiotics such as amoxicillin and enzyme inhibitors	Amoxicillin + Clavulanic Acid, Clindamycin, Metronidazole	Retrospective analysis at University Dental Clinical Center of Kosovo (2019–2022)
Tolaj, I. [18,19]	All COVID-19 patients in secondary healthcare hospitals were treated with antibiotics.	Ceftriaxone, Co-Amoxiclav	Cross-sectional study with 460 patients using the ID-IRI questionnaire
Mustafa, L [23]	ICU patients were treated with broad-spectrum antibiotics, raising concerns about resistance.	Imipenem, Ceftriaxone	Observational study of 52 ICU patients at University Hospital in Pristina
Aliaga, [5]	Ongoing study focusing on medication adherence during the pandemic	Various	Survey distributed among healthcare professionals and patients
Chandrasekara [29]	17% decrease in prescriptions during the pandemic.	Amoxicillin, Clindamycin	Retrospective data collection from urgent dental care records.
Subramanya [27]	AMR worsened due to disrupted global health programs and increased antibiotic use.	Various AMR-related drugs	Systematic review of AMR and antibiotic use during the pandemic.
Haliti [30]	Patterns of antibiotic utilization in Kosovo dental clinics	Amoxicillin, Clindamycin	Retrospective study at University Dentistry Clinical Center of Kosovo (2013)
Khami [31]	3.39-fold increase in self-medication with antibiotics post-pandemic.	Self-prescribed antibiotics, mainly broad-spectrum	Cross-sectional study of patient records pre- and post-pandemic.
Rabie & Figueiredo [32]	66% increase in prescriptions, especially antibiotics and analgesics.	Amoxicillin, Clindamycin, Ibuprofen	Retrospective analysis of prescriptions in public dental clinics.
Dar-Odeh [33]	Dentists are restricted to emergency treatments, raising concerns over AMR.	Various, including amoxicillin and clindamycin	Literature review on dental practices during COVID-19.
Rodríguez-Fernández [34]	Decrease in prescriptions during lockdown, but rebound in 2021.	Amoxicillin, Clindamycin	Quasi-experimental study on prescription data.
Bordea [35]	Emphasized the need for strict infection control to prevent virus spread.	Various dental-specific antibiotics	Systematic review of global dental policies during COVID-19.
Immel [16]	Significant reduction in antibiotic prescriptions during lockdown.	Amoxicillin, Ibuprofen	Retrospective time-series analysis of prescriptions.
Kitano [15]	31.2% reduction in antibiotic prescriptions during the pandemic.	Respiratory antibiotics	Interrupted time series analysis of outpatient prescriptions.
Aliaga [5]	Medication adherence worsened during the pandemic; findings are still ongoing.	Various adherence-related medications	Comprehensive survey of healthcare professionals and patients in Kosovo.
Soleymani [36]	Decreasing trends in dental antibiotic use reversed during COVID-19.	Penicillin, Clindamycin	Scoping review across global databases.
Cakolli [37]	High antibiotic use among dental students during COVID-19 in Kosovo.	Amoxicillin, Clindamycin	Survey of knowledge, attitude, and perception among dental students.
Petrac [38]	Consistent prescribing patterns over 5 years, but COVID-19 disrupted practices.	Amoxicillin, Clindamycin, Cefuroxime	Retrospective cohort study of national dental practices.
Sović [39]	Patterns of antibiotics used for endodontic therapy during COVID-19.	Amoxicillin, Clindamycin	Retrospective analysis of endodontic records during the pandemic.
Khan [40]	Increase in antimicrobial consumption in COVID-19 patients globally.	Azithromycin, Doxycycline	Systematic review and meta-analysis.
Ivanovic & Jokic [41]	Private dental organizations faced significant challenges during COVID-19.	Amoxicillin, Azithromycin	Observational study of private dental practices during the pandemic.
Sulis [26]	Widespread antibiotic abuse in low- and middle-income countries worsened by COVID-19.	Amoxicillin, Azithromycin	McGill University study on global antibiotic abuse trends.
Petrač [42]	Antibiotic consumption increased in Slavonia during the pandemic.	Amoxicillin, Penicillin	Observational study on antibiotic use in Croatian counties.
Serretiello [43]	AMR in Pseudomonas aeruginosa worsened during the pandemic.	Carbapenems, Fluoroquinolones	Retrospective cohort analysis in Italian hospitals.
Aliaga [5]	Survey study on medication adherence in Kosovo during COVID-19.	Amoxicillin, adherence-related medications	Survey conducted with healthcare professionals and patients in Kosovo.
Mancini [44]	Orthodontic emergencies and antibiotic use increased during COVID-19.	Amoxicillin, Doxycycline	Retrospective cohort study on orthodontic emergencies.
Abubakar & Sartelli [2]	Identified 10 golden rules for antibiotic use in hospital settings.	Broad-spectrum antibiotics	International call to action on optimizing antibiotic use in hospitals.
Haliti [22]	Over-prescription of antibiotics in Kosovo’s dental clinics.	Amoxicillin, Clindamycin	Retrospective analysis of dental prescriptions at the Oral Surgery Department.
Haliti [45,46]	Study on antibiotic utilization at the university dental clinic in Kosovo.	Amoxicillin, Clindamycin	Open Journal of Stomatology study on prescription trends.
Horvat [47]	Survey on knowledge and attitudes towards antibiotic use among prescribers.	Amoxicillin, Penicillin	Prospective antibiotic prescriber survey in Serbia.
Lila [48]	Pseudomonas aeruginosa prevalence and AMR trends in Kosovo’s University Hospital.	Carbapenems, Fluoroquinolones	Molecular epidemiology study at the University Clinical Center of Kosovo.
Etana [49]	Cross-sectional study on antibiotic use patterns at a dental clinic.	Amoxicillin, Metronidazole	Retrospective analysis of prescriptions in Ethiopian dental practices.
Hamiti-Krasniqi [50]	Local application of clindamycin reduced dry socket after molar extraction.	clindamycin	Randomized, placebo-controlled trial in Kosovo dental clinics.

Source: author collection from literature review.

## Data Availability

The data presented in this study are openly available in Preprints.org at https://www.preprints.org/manuscript/202501.0509/v1 (accessed on 24 March 2025). Additional data supporting the findings of this study are available upon request from the corresponding author. The data are not publicly available due to privacy and ethical restrictions.
